# Supplementary material for: The discovery of novel noncoding RNAs in 50 bacterial genomes
Source: Nucleic Acids Res. 2024 Apr 22;52(9):5152–65. doi: 10.1093/nar/gkae248 (PMC11109978; doi:10.1093/nar/gkae248)
Supplement: gkae248_Supplemental_Files [file gkae248_supplemental_files.zip › Narunsky 50 Genomes RNA Motifs Files.zip!/Publication/61-Geobacter_sp_M21/Figures/LRC-61-26-NC_012918_1_IGR_93_109922_110399.sto.pdf]

LRC-61-26-NC_012918_1_IGR_93_109922_110399.sto.r2r

5′-○○○○○○○○○Y●G●CCGCCGCUCYC●●○○G●GCGG●●●UUUUYYRUUUUUUGACGCG●CCG●GA●G●GCYUC○●YCRU○○GUGACG●UAUUUGAC●C●○YUYCR○●GG●U●U●●●CY●A●RU●CY●AYU●UY●G●CGUUU●CYUGRY●○○AYC●●●●●Y●●●U●AR●●●YGYU●AU●AGAGCUGU●●YR●GCC●○○○AAURA○○○○○○○ARAU●UUUUCGG●G●AU●CR●CY●C●GYY●U●●UR●●●●U●AY●Y●●C●●U●AUU●●RR●A●●A●●●YG○GRCC●G●C●Y●GCY●U●C●G●GR●G○○○●CGG○GC●GCGR○○○○●GRAUU●C●YR●●●A●●YRRRY○●●YG●●RAUUAR●●●GGCACGCA●GGUG●A●●AC●●ACUCCCYAUCGGCCGCAG○○○●AAAAAAACCGGCRAGAA●●C●GR○●CAA○AACCAGAARGGGGAR○○○○○
